# A Case of Primary Lung Adenocarcinoma With Metastasis to Colon Harboring EGFR Exon 19 Deletion

**DOI:** 10.7759/cureus.63665

**Published:** 2024-07-02

**Authors:** Zhongqian Lin, Frederic Karim, Aaron Yee

**Affiliations:** 1 Internal Medicine, New York-Presbyterian Brooklyn Methodist Hospital, Brooklyn, USA

**Keywords:** immuno-histochemical, genomic analysis, adenocarcinoma of colon, pulmonary adenocarcinoma, metastasis from primary lung, egfr exon 19 mutation

## Abstract

Lung cancer metastasizing to the colon is exceedingly rare and can present similarly to colorectal cancer. It is crucial to conduct further evaluations using immunohistochemical (IHC) stains and genomic testing to differentiate between the two and provide appropriate treatment without delay. Lung cancer generally has a poor prognosis, especially in cases with distant metastases. Although gastrointestinal (GI) metastases from lung cancer have been reported, cases of lung cancer manifesting as colon metastasis are extremely rare, with only a few instances documented.

## Introduction

Lung cancer is the leading cause of cancer death among both men and women in the United States. In 2023, an estimated 132,330 Americans died from lung cancer, representing approximately 22% of all cancer deaths [1]. Common metastatic sites include the bone, brain, adrenal glands, and liver. The gastrointestinal (GI) tract is a less frequent site of metastasis, predominantly involving the jejunum or ileum [2]. Colonic metastases are particularly rare, with an incidence of about 0.1% [3]. EGFR exon 19 deletion has been reported in only two lung cancer patients with GI metastases, both of which were squamous cell carcinomas [4]. Here, we present an unusual case of primary lung adenocarcinoma with colon metastasis harboring EGFR exon 19 deletion, initially presenting with GI bleeding.

## Case presentation

**Figure 1:**
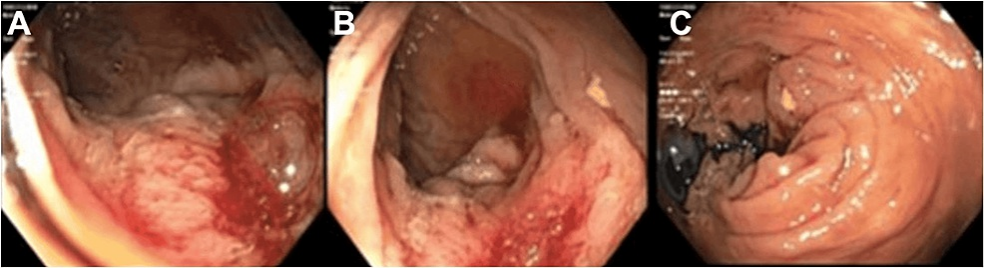
Endoscopic findings of advanced tumor lesions in the (A,B) ascending colon with a view from the (C) hepatic flexure.

**Figure 2:**
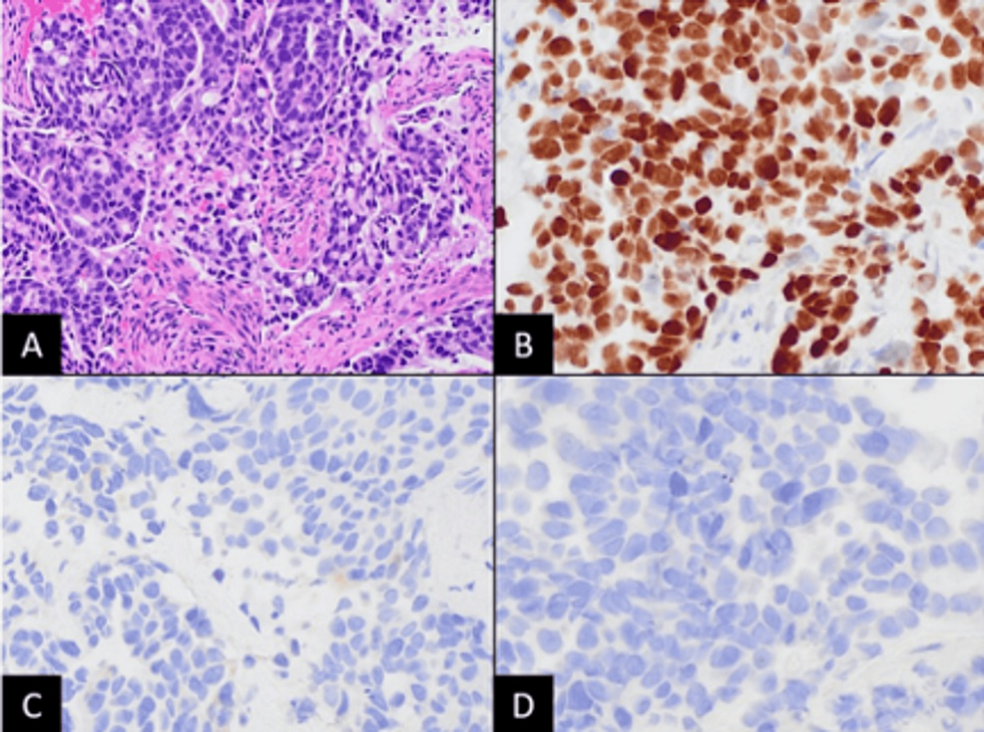
(A) Endoscopic biopsy of the colonic mass revealing high-grade adenocarcinoma (hematoxylin-eosin staining, 100× magnification). (B) Immunohistochemical staining showing that the tumor cells are immunoreactive to thyroid transcription factor-1 staining. (C) Negative staining for primary colonic tumor markers, cytokeratin 20. (D) Negative staining for caudal-type homeobox-2.

**Figure 3:**
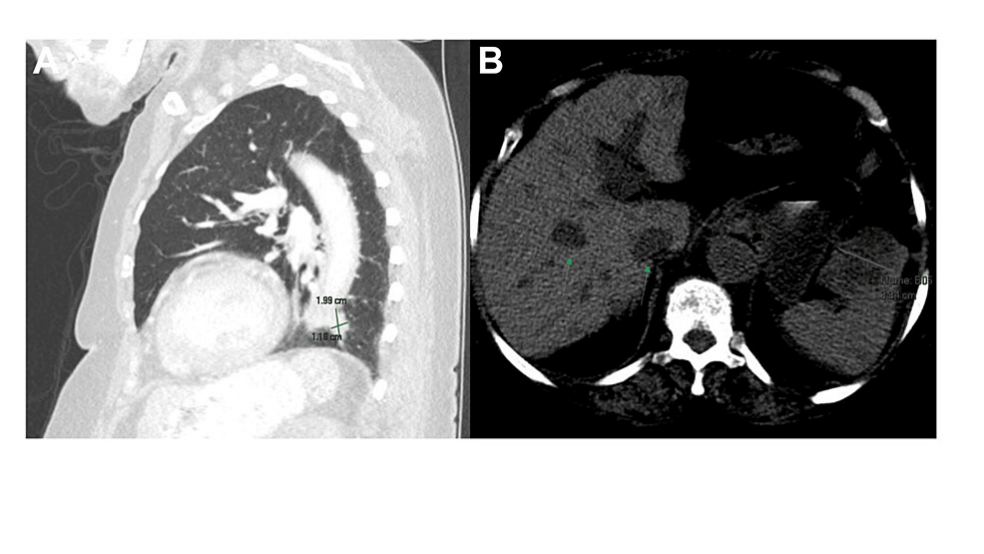
CT scan of chest, abdomen, and pelvis revealed (A) pulmonary mass in the left lower lobe and (B) hepatic masses.

**Figure 4:**
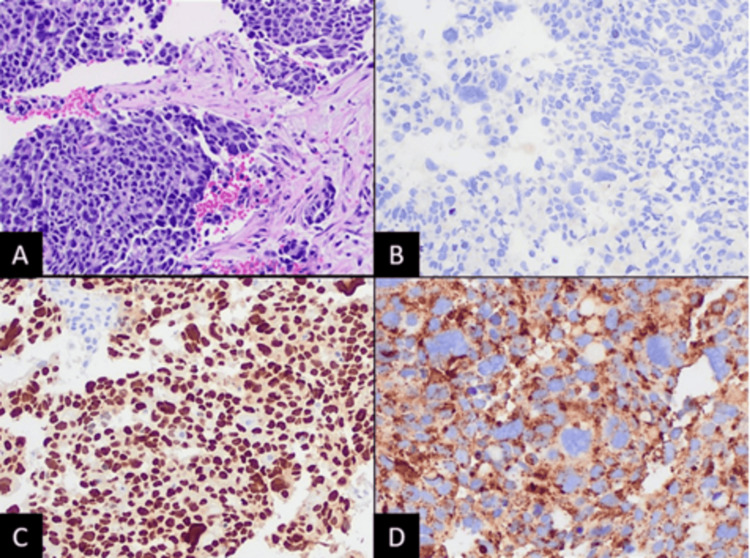
(A) CT-guided core needle biopsy of the liver mass showing neoplastic proliferation of atypical cells associated with a desmoplastic reaction (hematoxylin-eosin staining, 100× magnification). Staining of the neoplastic cells: (B) negative for glypican-3, (C) positive for thyroid transcription factor-1 (nuclear staining), and (D) positive for napsin-A (coarse granular cytoplasmic staining, 200× magnification).

A 71-year-old female with a past medical history of hypertension, hyperlipidemia, and type 2 diabetes mellitus, but no prior oncological history, presented with several weeks of melena, constipation, and constitutional symptoms, including malaise, anorexia, and unintentional weight loss. Her vital signs were normal, but a physical examination revealed an elderly female with fatigue, dry mucous membranes, poor skin turgor, and dark, soft stool on a digital rectal exam. Laboratory results are listed in Table 1. She was initially evaluated by gastroenterology for the melena and underwent a colonoscopy, which identified a tumor-like lesion in the ascending colon (Figure 1). Biopsy of the ascending colon lesion revealed moderately differentiated adenocarcinoma (Figure 2). A CT scan of the chest, abdomen, and pelvis with intravenous contrast revealed diffuse metastatic lesions in the lungs, thoracic lymph nodes, liver, spleen, and bone, with epidural extension of disease at the S1 and S2 segments, which the patient was previously unaware of (Figure 3). A liver biopsy confirmed adenocarcinoma (Figure 4).

**Table 1:** Initial laboratory results

Laboratory test	Value	Reference range
Hemoglobin	6.5 g/dL	11.6-15 g/dL
White blood cell	15,400/mm^3^	4,500-11,000/mm^3^
Alkaline phosphatase	164 IU/L	44-147 IU/L
Aspartate transaminase	71 U/L	8-33 U/L
Alanine transaminase	16 U/L	4-36 U/L
Total bilirubin	1.5 mg/dL	<1 mg/dL
Serum carcinoembryonic antigen	4,343 ug/L	≤3.0 ug/L

**Figure 5:**
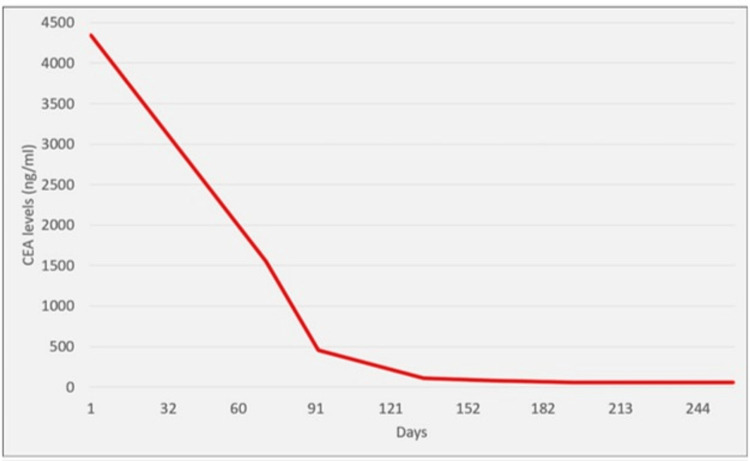
Trend of CEA levels over time

One month later, the patient was readmitted for hematochezia and a hemoglobin level of 5.7 mg/dL. She was then stabilized and improved with red blood cell transfusion. FOLFOX therapy was initiated and completed for the suspected ascending colon adenocarcinoma during this hospitalization. Four days post-discharge, IHC stains from both colon and liver biopsies revealed cancer biomarkers positive for CK7 and TTF-1 and negative for CK20, CDX2, PAX8, and GATA3, strongly suggesting metastasis from primary lung adenocarcinoma (Figures 2, 4). Tumor somatic mutation analysis from a peripheral blood sample using Guardant360 showed EGFR exon 19 deletion, with a high allelic frequency of 25%, EGFR amplification, and ESR1 mutation. Next-generation sequencing on both liver and colon biopsies confirmed the EGFR exon 19 deletion in the colon specimen. The patient was subsequently initiated on osimertinib and showed a positive response with downtrending CEA levels (Figure 5). A follow-up CT scan showed a marked decrease in the size and number of hepatic and splenic lesions.

## Discussion

Metastatic disease of the colon from primary lung cancer is exceedingly rare and underreported, though increasingly recognized due to advances in treatment and longer patient survival. The clinical incidence of GI metastases from primary lung cancer is estimated at 1.7% [2], while autopsy studies suggest a prevalence ranging from 4.7% to 14% [5-7], indicating occurrence primarily in advanced stages and likely underreported clinical incidence due to asymptomatic metastatic disease, non-specific symptoms, and diagnostic challenges. GI metastases from lung cancer confer a poor prognosis, with a median overall survival of 2.8 months and 53.4% mortality within three months of diagnosis [8]. Colonic metastasis is rare, with an incidence of 0.1%. The small bowel is the most common site of metastatic lung cancer in the GI tract [2,8,9], likely due to its abundant blood supply and higher potential for serious complications, such as perforation, obstruction, or bleeding [10].

Previous retrospective studies with small sample sizes suggest that GI metastases from primary lung cancer occur mostly in elderly men, with adenocarcinoma being the predominant histological subtype in some studies [10-12], while others suggest that large cell carcinoma and squamous cell carcinoma are more common [5,6,8]. Adenocarcinoma is associated with a lower risk of perforation compared to large cell and squamous histologies [8,13].

Only two cases of primary lung cancer with GI metastases harboring EGFR exon 19 deletion have been reported, both of which were squamous cell carcinomas, and one responded transiently to erlotinib [4]. Our patient also had EGFR exon 19 deletion and responded well to osimertinib. Whether EGFR exon 19 deletion is associated with an increased risk of GI metastases remains unclear.

Endoscopic evaluation and imaging studies are useful in diagnosing GI metastases in known lung cancer patients. However, differentiating primary colon cancer from metastatic lung cancer poses a diagnostic challenge when lung cancer presents primarily as a GI tract mass. Positive IHC staining for thyroid transcription factor 1 and cytokeratin-7 and negative staining for CK20 and CDX2 strongly correlate with GI metastases from lung cancer (Figure 2).

Common metastatic sites for lung malignancy, which includes non-small cell lung cancer (75.3%) and small cell lung cancer (24.7%), are bone (41.4%), contralateral lung (32.8%), liver (23.9%), brain (27%), and adrenal glands (19.5%) [14]. Lung cancer has the highest mortality rate worldwide and is the leading cause of cancer deaths, reflected in low survival rates. The overall five-year survival rate is 55.2% for patients with localized disease, but only 16% of patients are diagnosed at an early stage [15]. Early treatment post-diagnosis is beneficial, as even a one-month delay increases the risk of death by up to 13%. Cancer biomarkers and genomic testing from biopsies guide treatment options, but the turnaround time for these results can be delayed by weeks to months. In our patient, the initial presentation resembled primary colorectal malignancy, leading to the initiation of FOLFOX therapy. However, upon receiving IHC and genomic testing results, the treatment was appropriately modified.

## Conclusions

Lung cancer metastasizing to the colon is exceptionally rare and can present similarly to colorectal cancer. Performing IHC and genomic testing is crucial to distinguish between the two and provide appropriate treatment. Lung cancer has a generally poor prognosis, particularly in patients with distant metastases. Physicians should weigh the potential increased turnaround time for biomarker finalization against the benefits of early therapy initiation to improve patient survival. Although GI metastases from lung cancer have been reported, cases of lung cancer manifesting as colon metastasis remain extremely rare.
